# Accounting for multiscale processing in adaptive real-world decision-making via the hippocampus

**DOI:** 10.3389/fnins.2023.1200842

**Published:** 2023-09-05

**Authors:** Dhruv Mehrotra, Laurette Dubé

**Affiliations:** ^1^Integrated Program in Neuroscience, McGill University, Montréal, QC, Canada; ^2^Montréal Neurological Institute, McGill University, Montréal, QC, Canada; ^3^Desautels Faculty of Management, McGill University, Montréal, QC, Canada; ^4^McGill Center for the Convergence of Health and Economics, McGill University, Montréal, QC, Canada

**Keywords:** hippocampus, reinforcement learning, successor representation, decision making, self

## Abstract

For adaptive real-time behavior in real-world contexts, the brain needs to allow past information over multiple timescales to influence current processing for making choices that create the best outcome as a person goes about making choices in their everyday life. The neuroeconomics literature on value-based decision-making has formalized such choice through reinforcement learning models for two extreme strategies. These strategies are *model-free* (MF), which is an automatic, stimulus–response type of action, and *model-based* (MB), which bases choice on cognitive representations of the world and causal inference on environment-behavior structure. The emphasis of examining the neural substrates of value-based decision making has been on the striatum and prefrontal regions, especially with regards to the *“here and now”* decision-making. Yet, such a dichotomy does not embrace all the dynamic complexity involved. In addition, despite robust research on the role of the hippocampus in memory and spatial learning, its contribution to value-based decision making is just starting to be explored. This paper aims to better appreciate the role of the hippocampus in decision-making and advance the successor representation (SR) as a candidate mechanism for encoding state representations in the hippocampus, separate from reward representations. To this end, we review research that relates hippocampal sequences to SR models showing that the implementation of such sequences in reinforcement learning agents improves their performance. This also enables the agents to perform multiscale temporal processing in a biologically plausible manner. Altogether, we articulate a framework to advance current striatal and prefrontal-focused decision making to better account for multiscale mechanisms underlying various real-world time-related concepts such as the self that cumulates over a person’s life course.

## 1. Introduction

After a long day at work, it is time to go home. If one has worked in the same building for several years, one does not actively think about how to get out of the building as a key milestone on the way to the goal of getting home. This action simply involves coming out of the elevator and turning left or right to exit onto the street. This simple decision can be a bit different, though, if construction in the building blocks the exit. Instead of getting out of the elevator as usual, one may remember a nearby fire exit and get out from the building.

This extremely simplistic example of a real-world behavior sequence in reaching a goal has been used in previous psychological research in human decision-making (Kruglanski and Szumowska, 2020; Wood et al., 2021). Clearly, making a choice with the best outcome is more complex than this simplistic example most of the time. It is also a lifelong challenge that requires considering outcomes on different timescales and calling for adaptation to stable, diverse, and changing contexts that one encounters every day and over one’s life course (Decker et al., 2016).

Examining adaptive behavior from such a lifespan behavioral and decision neuroscience perspective calls upon the interface of value-based decision-making with literature on learning, memory, and spatial navigation (Johnson et al., 2007). The literature on value-based decision making has formalized two types of reinforcement learning strategies used in decision-making: *model-free* (MF), which is an automatic, stimulus–response type of action. The other type of strategy is called *model-based* (MB), wherein we use the knowledge of the cognitive representation of the world around us and causal environment-behavior inference to plan our next action with more flexibility but also less efficiency. MB action is an important component of planning and deliberative decision-making, where one needs to mentally imagine future scenarios and make a choice, something which we routinely do in our lives. MB ability has shown a developmental pattern, progressively emerging with age from childhood to adulthood (Decker et al., 2016).

Broadly speaking, the MF and MB strategies have been attributed to distinct brain regions; the striatum is involved in automatic responses which are the hallmark of MF strategies, while the hippocampus is thought to be key not only for episodic and spatial memories but also for building a model of the world.

The striatum is associated with the dopaminergic system and reward, as well as neural representations that track value. These properties have resulted in the striatum becoming a hotbed of focus for researchers studying neuroeconomics, decision-making and behavioral neuroscience. Fundamental studies in psychology (Pavlov, 1960; Kamin, 1969) have lent themselves well to quantitative approaches, facilitating the growth and emergence of several computational models of reinforcement learning (See Samson et al., 2010 for review). Computational models of deliberation and planning in the brain are relatively more recent (Mattar and Lengyel, 2022; De Martino and Cortese, 2023), and consequently the interactions between the hippocampal and striatal system have only recently garnered attention.

For example, Ferbinteanu (2020) showed that the hippocampus supports both spatial and habitual memories when these events have temporal proximity, while the striatum supports both types of memories for events sharing a common spatial context. Models unifying the two systems have also been presented in relation to decision-making. Geerts et al. (2020) introduced a model in which the hippocampal-striatal system was viewed as a general system for decision making via an adaptive combination of the MF and MB frameworks. However, more research is needed to better understand the dynamics of interaction between the two systems, especially in real-world contexts, which are ever-changing (Goodroe et al., 2018).

Taken together, there is a need to move beyond the present false dichotomy implying that value-based decision making is either MF or MB and better appreciate the role of the hippocampus in decision-making, beyond its role in the encoding of episodic memories (Scoville and Milner, 1957). This is exemplified by RL models using replay as a strategy to improve task performance (Russek et al., 2017; van de Ven et al., 2020). The hippocampus too exhibits replay, suggesting that it could be contributing to reward learning in the brain. Understanding this crucial link opens avenues to understanding hippocampal contributions to decisions in real-world timescales as well as long-term decisions as episodes accumulate over a person’s life course, impacting an individual’s health and overall wellness.

In the subsequent sections, we will review RL models and how they inform our thinking about neural processes. In relation to these models, we will introduce the hippocampus as a sequence generator (Buzsáki and Tingley, 2018). We will review specific examples of hippocampal sequences to demonstrate that these sequences can be used for MB actions. While most of this work has been done in the rodent spatial navigation system, the prevailing notion is that these sequences are attributed with meaningful content as the animal experiences its environment (Buzsáki and Tingley, 2018). Thus, hippocampal sequences can be generalized to any form of multimodal information that is sequential in nature. Studying spatial navigation simply provides a convenient, tractable foray into understanding hippocampal function. Specifically, we will argue that the hippocampal provides key neural substrates for the continuous sense of the self over time along a person’s life course. Finally, we will suggest potential ideas for interdisciplinary convergence bridging animal systems to human neuroscience studies as well as to the fields of neuroeconomics.

## 2. RL models of decision-making

The main aim of RL models is for an agent to maximize its reward given a state and learn the optimal action policy for it to do so. For example, these states could be locations, stimuli, or reward contingencies. RL agents broadly fall into two categories: MF and MB. MF agents learn via prediction errors between the expected value of the state and the observed value, a process known as temporal difference (TD) learning. These agents try to minimize the prediction error between observed and expected reward value over the long term. This approach is typically computationally faster and cheaper, but such agents have no memory of past states or the relationship between them. For example, if the value of the reward changes, a MF agent will only be able to update itself by revisiting different states several times and experiencing the consequences of its actions repeatedly.

On the other hand, MB agents learn a representation of the various states and transitions between them and use this to maximize long-term expected reward value. Representing the entire set of transitions and states is what makes these models flexible to novel task demands. Unlike a MF agent, a MB agent would not need to experience states repeatedly to learn changes in reward values; the process is much faster since the agent is able to exploit the task structure to learn such changes. However, MB algorithms are significantly more computationally intensive.

RL approaches lend themselves well to biology and provide a framework to generate testable predictions about the working of the striatal system in the context of reward learning and decision-making. In the brain, the striatum is thought to signal value, which is updated by a dopaminergic prediction error signal (Schultz et al., 1997), and these dopamine responses are in line with predictions of TD learning models (Waelti et al., 2001).

How the brain learns the model of the environment is a relatively more complex question to tackle. Unlike MF approaches which emphasize stimulus–reward associations, animals do not even need reward to learn the structure of the environment. The retention of information in the absence of external reinforcers is referred to as *latent learning* (Tolman, 1948). Latent learning enables the animal to quickly predict future rewards, or even generalize learnt knowledge to other state spaces. Such mental representations of the environment came to be known as *cognitive maps*. How cognitive maps contribute to MB agents remains a gap in the field. In the 1970s, the discovery of place cells (O’Keefe, 1976) brought the hippocampus into focus as the seat of the cognitive map. More recent studies in humans have begun to show the direct contributions of the hippocampus to MB planning (Miller et al., 2017; Vikbladh et al., 2019). Therefore, understanding hippocampal function is an essential avenue to further our knowledge of MB decision-making.

## 3. The successor representation

Current experimental setups sometimes fail to accurately assess how and to what extent an agent uses MF and MB strategies in decision-making problems, and a re-evaluation of the assumptions underlying these strategies is much needed (Feher da Silva et al., 2023). This would then allow for further investigation into the role of the hippocampus within MF and MB actions more clearly.

Therefore, more recent methodologies in RL emphasize a combination of MF and MB agents to improve the generalization of TD learning approaches. One such approach that has gained popularity in neuroscience is the successor representation (SR) (Dayan, 1993; Gershman, 2018).

RL provides a formal means of investigating decision-making, in which states of the world are rewarded and decisions must be made on the selection of actions that can be taken to maximize reward. In this framework, each state has a value (*V*), defined as the cumulative expected reward over future states, multiplied by a discount factor (γ∈(0,1)) that reduces the weight of distal rewards.

It is useful to make decisions based on the estimated value of different states. As shown by Dayan (1993), the value function can be mathematically represented as the inner product of the reward function (*R*) and a representation of the estimated value of states (*M*), as shown below:


(1) $$V\left(s\right)=\underset{s\prime }{\sum }M\left(s,s^{\prime }\right)\;R\left(s^{\prime }\right)$$

The matrix *M* is the SR matrix. The SR possesses a state representation which conveys the discounted number of expected visits of a given future state (*s*’) from a given starting state (s). The SR matrix is given by:


(2) $$M=\overset{\infty }{\underset{t=0}{\sum }}\gamma^{t}T^{t}$$

**Figure 1:**
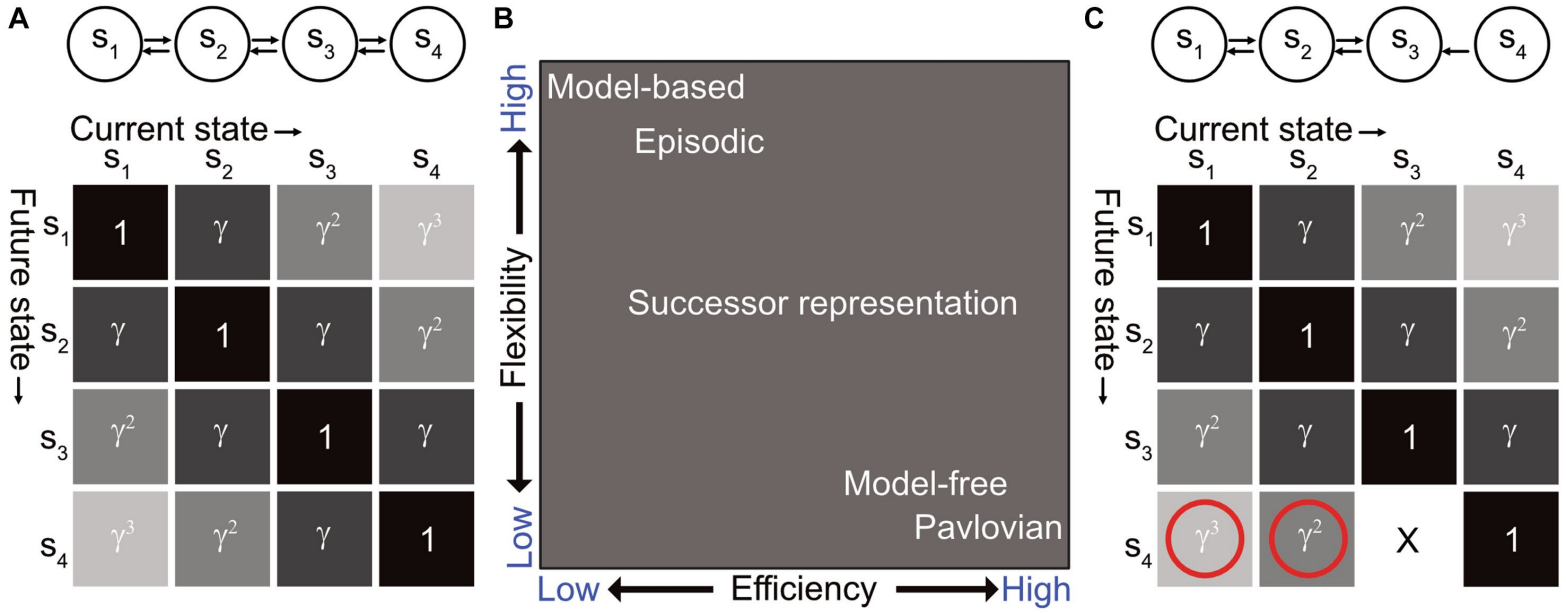
**(A)** (Top) Illustrative example of an agent traversing between 4 states s_1_ to s_4_, the relationship between which is depicted by the arrows corresponding to allowed state transitions. (Bottom) The successor matrix for this state diagram, where γ depicts the discount factor. Under a random walk policy, each column of this matrix has a value of 1 on its diagonal and gradually decreasing in either direction. In terms of occupancy relative to other states, these columns resemble hippocampal place fields. **(B)** Comparison between different RL agents for the efficiency-flexibility trade-off. The efficiency axis represents the degree to which the model requires costly versus cheap computation. The flexibility axis represents the degree to which the agent can adapt flexibly to changes in the environment, i.e., how much new data needs to be gathered for value estimates to converge to the correct value. Figure adapted from Gershman (2018). **(C)** The same SR matrix as shown in panel **(A)**, but for a task where now there is no more state transition from s_3_ to s_4_. This is an example of a transition revaluation. In this case, the SR can correctly update the changed transition from s_3_ to s_4_ but is unable to update the entries preceding this transition, i.e., the transitions from s_1_ to s_4_ and s_2_ to s_4_ should also go to 0.

Where T is the transition matrix, and t denotes all future time steps in the planning horizon. Instead of computing the transition matrix for each step, the SR is computed as a discounted sum going from state s to state s’ in a given number of steps, determined by the planning horizon (Figure 1A). This representation therefore has predictive structure, akin to a MB agent, but can be learned by a MF agent via TD learning, by learning the difference between observed and expected state occupancy. The SR approach thereby integrates the advantages of a MB agent into a MF framework (Figure 1B).

From Equation (1), we observe that the SR is a representation of possible future states that can be separable from the value function. In a reward revaluation task (i.e., change in reward value), this allows the agent to retain the same predictive map and quickly compute the value, whereas an MB agent would have to recompute the mapping between states, and an MF agent would have to re-learn the environment altogether. The SR thus offers an optimal solution to this kind of task and permits the learning of the state transitions (or “map”) independently of reward.

Additionally, Equation (1) also provides a direct relationship between the value function and SR, suggesting that updates to SR can update the value function. The SR, therefore, forms an important link between predictive representations and the value-based decision-making framework.

The SR, however, has its own set of caveats. It requires direct experience to learn, akin to an MF agent. If the transition structure between states were to change through the course of the task (known as transition revaluation), the SR would only be able to update the one-step transition but not the steps preceding this state (Figure 1C). This is because the SR is probabilistic and has no temporal representation built into it. As a result, SR agents are unable to solve transition revaluation or policy revaluation (i.e., change in strategy) tasks, which animals can easily adapt to Tolman (1948) and Simon and Daw (2011).

Despite the caveats, SR models have been of increasing interest in neuroscience due to their biological plausibility, accompanied with observations of their behavioral and neural correlates during decision-making. Using a sequential learning task (Momennejad et al., 2017), human participants learnt a relationship between stimulus and reward, which was manipulated in the re-learning phase, and subsequently probed in the final phase of the task. In the re-learning phase, the investigators performed either a reward revaluation, or a transition revaluation. As detailed above, an SR agent would be able to solve reward revaluation but not transition revaluation. This was recapitulated with the participants; they were able to adjust better to reward revaluation compared to transition revaluation, suggesting the utilization of cached representations (analogous to SR) to solve the task.

In addition, the SR has neural correlates in the hippocampus: If a SR agent is allowed to forage in an open arena with uniformly distributed rewards and there exists a population of neurons encoding each spatial state, the neural population activity (i.e., the columns of the SR matrix) resembles hippocampal place fields (spatial locations where cells fire most, in an arena) (Stachenfeld et al., 2017). In the same study, the authors showed that the eigenvectors of the SR matrix resemble grid cells (cells that fire in a hexagonal grid-like pattern within a given environment). Predictions from such models also recapitulated experimental observations such as the clustering of place fields around rewarded locations (Hollup et al., 2001) and the distortion of place fields around barriers and other environmental distortions (Muller and Kubie, 1987; Skaggs and McNaughton, 1998; Alvernhe et al., 2011). Furthermore, the emergence of place cell and grid cell activity itself has been shown to emerge via learning of a SR agent that uses boundary vector cells (cells that respond to a boundary in the arena, at a particular distance and direction from an animal) as the fundamental unit of spatial representation (de Cothi and Barry, 2020).

Recent work on the SR has tried to address and resolve the lack of temporal resolution in the SR. Momennejad and Howard (2018) showed that an ensemble of SR matrices with different discount factors (denoting different timescales) can be used to incorporate sequential order by encoding the Laplace transform of the future. A Laplace transform decomposes a signal into exponential decay functions of different rates. The inverse of this is equivalent to computing a derivative of the relation between two given states across SR matrices, i.e., across timescales. This consequently enables recovery of the temporal order between states. The mathematical formulation of this approach resembles that used in the temporal context model, detailed in a later section (See section: A broader view of hippocampal sequences).

A prediction that arises from multi-scale SR is the presence of cells that are sequentially activated as a function of the distance to the goal (Momennejad and Howard, 2018). Such cells have been experimentally observed in the hippocampus of bats and mice (Sarel et al., 2017; Gauthier and Tank, 2018), as well as in the human entorhinal cortex, which is the principal input and output structure of the hippocampus (Qasim et al., 2018). Interestingly, these goal-vector cells are also compatible with path-integration models of the hippocampus, in which they permit rapid generalization of policy (Whittington et al., 2022).

Additional support for multi-scale SR in the brain, comes from a study by Brunec and Momennejad (2022), where they analyzed functional magnetic resonance imaging (fMRI) data collected from human participants completing a virtual navigation task and analyzed predictive horizons in the hippocampus and prefrontal cortex (PFC). Briefly, a predictive horizon is a measure of how far ahead into the future is predicted by the activity in these brain regions. Long predictive horizons correspond to longer-range planning. The authors found that predictive horizons in the hippocampus followed an anatomical gradient. This anatomical gradient is consistent with a gradient of increasing place field sizes in the hippocampus (Jung et al., 1994; Kjelstrup et al., 2008), suggesting a temporal role for the anatomical gradient. This result emerges independently in the multi-scale SR model of George et al. (2023), where the authors show multi-scale SR being stored by differently sized place fields, but only when these place fields are segregated along an anatomical gradient. These findings are in line with the forgetting of recent experiences during hippocampal lesions, as in the case of H.M. (Scoville and Milner, 1957).

Interestingly, predictive horizons analyzed in Brunec and Momennejad (2022) were always larger in the PFC compared to the hippocampus. Indeed, the orbitofrontal cortex may be involved in the representation of task and state spaces (Wilson et al., 2014; Wikenheiser and Schoenbaum, 2016). These findings suggest that neural correlates of the SR may also be found in regions other than the hippocampus, but especially those which are in close association with the hippocampus. The gradient of predictive horizons in the hippocampus and PFC is also in line with intact past experiences during hippocampal lesions (Scoville and Milner, 1957), corroborating the hypothesis that the hippocampus is a temporary storage for memories until they become consolidated in the cortex, known as *systems consolidation*.

Taken together, these observations provide evidence for the utility of SR in using RL-based approaches to understand the neural representations of space in the brain and more directly exhibit the predictive nature of hippocampal representations (Stachenfeld et al., 2017), suggesting that a multi-scale SR might be implemented across brain regions, spanning the hippocampus to the PFC, warranting further investigation into the mechanisms behind how these regions communicate during real-world decisions.

In summary, the SR is an RL-based framework of predictive representations that combines some of the speed of MF and the flexibility of MB agents. Such a predictive system is reminiscent of the hippocampal memory system, as evidenced from various neural correlates of the SR in the hippocampus. Most models of hippocampal function focus on learning (Uria et al., 2020; Whittington et al., 2020; George et al., 2021) and memory (Marr, 1971; Teyler and DiScenna, 1986; Spalla et al., 2021). While there are some models of the hippocampus that also learn via prediction (Uria et al., 2020; Whittington et al., 2020), they do not directly provide insights into how these predictions inform value-based decisions. The SR is unique in that it directly links prediction to value, thereby providing a platform to better understand hippocampal contributions to the extensively studied field of value-based decision-making.

## 4. Linking SR models to hippocampal sequences

The SR being a state-based model relies on the delineation of explicit states that the agent can be in at any given time. In a computational agent, these states are explicitly encoded. However, if animals were to implement the SR, these states are likely learned and updated from experience. The learning of the SR, therefore, is an interesting research direction that warrants future work that can potentially inform real-world decision-making.

Traditionally, SR models were learnt using TD learning, which is not known to be implemented in the hippocampal circuitry (but see Foster et al., 2000; Johnson and Venditto, 2015). Recently, a series of reports have demonstrated biologically plausible learning of the SR (Bono et al., 2023; Fang et al., 2023; George et al., 2023). These studies use different mechanisms to demonstrate SR learning, such as: (1) spike-timing dependent plasticity on temporally compressed trajectories called theta sequences (George et al., 2023) (See section: Theta Sequences and Prospective Coding in the hippocampus), (2) a plasticity rule on a spiking feed-forward network mimicking anatomical input to the hippocampus (Bono et al., 2023), and (3) a recurrent neural network with weights trained via an anti-Hebbian learning rule (Fang et al., 2023). These mechanisms are not mutually exclusive from each other, suggesting a degeneracy of candidate mechanisms for SR learning in the hippocampus. The SR could also be learnt from sequential models of the hippocampus (George et al., 2021), which can distinguish between aliased sensory observations.

Work on how the SR is learnt and updated has also given rise to models that perform better than classical SR models and provide not only a better understanding of hippocampal function, but also lend valuable insights into real-time decision-making in real-world contexts. Russek et al. (2017) introduced an SR agent that can solve transition and policy revaluation tasks, called SR-Dyna. This agent learns representations through online experience, and in addition prioritizes recent experience using “offline replay,” referring to the simulation of experiences by playing back past episodes (Lin, 1992). In addition, SR-Dyna operates over state-action pairs, in contrast to other SR agents which operate on states alone. In the absence of replay, SR-Dyna performs as well as a classical SR agent, thereby making it better than a MF agent. But with replay, SR-Dyna can perform exceedingly well, solving tasks that typical SR agents fail to solve.

Offline replay has been shown to be a key process in contributing to generalization and memory consolidation in several human and animal studies (Girardeau et al., 2009; Maingret et al., 2016; Momennejad et al., 2018; Schapiro et al., 2018; Liu et al., 2019). Replay can be thought to update the model of the environment via consolidation, and this updated model can be used for subsequent planning. An implementation of replay, therefore, was probably inspired by some of these studies. SR-Dyna replays experienced transitions to update the successor matrix, and the quantity of this replay is directly related to the performance of the agent, thereby providing quantitative insights into the influence of hippocampal replay on updating reward representations in the striatum, which in turn would optimize reward-guided behavior.

The existence of anatomical substrates for the integration of replay into the reward learning system (thought to be implemented by the striatum) makes SR-Dyna well-poised to further understand the role of hippocampus in decision-making. In particular, the hippocampus and the dopamine system form an anatomical loop; the hippocampus receives dopaminergic inputs from the ventral tegmental area (VTA) and in turn projects to the ventral striatum (nucleus accumbens) and globus pallidus, which projects back to the VTA (Lisman and Grace, 2005). This anatomical organization of the two systems suggests that not only can the hippocampus directly influence striatal function (Sjulson et al., 2018), but the dopamine system can also signal the relative valence of experiences to the hippocampus.

In summary, RL approaches to decision-making have provided a mathematical framework to understand how the brain can possibly implement reward learning, and thereby pursue the strategy that leads to maximal expected reward (Samson et al., 2010). However, we still lack a comprehensive understanding of how the brain learns the structure of the environment and implements MB algorithms for efficient decision-making. We propose that this gap in the field can be bridged by appreciating the role of the hippocampus in decision-making.

In subsequent sections, we will review further evidence for the role of the hippocampus in prospective coding, i.e., future planning of actions. To do so, we will further build upon hippocampal replay, which is a form of offline consolidation. We will then introduce a substrate for prospective coding known as theta sequences (Foster and Wilson, 2007), which is a form of online planning. Finally, we will extend concepts of spatial coding to the temporal domain, and touch upon large-scale brain networks involving the hippocampus.

## 5. Linking the successor representation to the memory system

In the 1940s, Tolman (1948) performed behavioral experiments with rats navigating a maze. Once the rats learnt the reward location, the shape of the maze was drastically altered. Yet, the rats were able to efficiently navigate to the same reward location, regardless of the shape of the maze. This observation suggested that the animals were able to form a representation of spatial location, without any direct stimulus–reward association, known as *latent learning*. This also begged the question of the neural correlates of the cognitive map that the animals used to reach the reward, long before the arrival of computational models of RL.

Fast-forward to the 1970s. With advances in electrophysiology, it became possible to record neurons in freely moving animals. This led to John O’Keefe’s discovery of place cells in the hippocampus (O’Keefe, 1976). This discovery generated interest in the hippocampus as the seat of the cognitive map. Subsequently, it was found that the hippocampus represents several other neural representations based on what is salient information for the task at hand, such as time (Pastalkova et al., 2008), a conspecific (Danjo et al., 2018), sound frequency (Aronov et al., 2017), value (Knudsen and Wallis, 2021), sensory evidence (Nieh et al., 2021), or past and present spatial trajectories (Frank et al., 2000; Wood et al., 2000), all of which can be encoded as potential states in an SR-based representation, with a discount factor that is dependent on how these variables change with time; a stable environment would have a larger discount factor. Taken together, this evidence suggests that the hippocampus is a key region for the representation of task-relevant information critical for SR models.

In summary, the prevailing theory of hippocampal function is thought to be the binding of spatial, temporal, and other sensory features into an episode, thereby being important for episodic memories (Buzsáki and Tingley, 2018; Whittington et al., 2022). SR models can provide a computational framework for episodic learning (Gershman et al., 2012), and have demonstrated that the representation of space and perhaps other variables of interest is not merely a static representation, but a predictive one, encoding the statistics of future expectations (Lisman and Redish, 2009; Stachenfeld et al., 2017).

## 6. Hippocampal replay

Once it became possible to simultaneously record several neurons in the hippocampus, researchers could now investigate the population activity of the hippocampus. Recording several place cells as a rat ran around in a freely moving arena, Wilson and McNaughton (1994) observed that neurons that tended to fire together when the animal was exploring an arena also tend to fire together during post-task sleep. Such reactivations usually occur during non-Rapid Eye Movement (NREM) sleep or during quiet wakefulness when the animal is disengaged from its environment, such as during grooming or consummatory behaviors (Carr et al., 2011). These reactivations are nestled within periods of elevated hippocampal population firing known as sharp-wave ripples (SWRs).

**Figure 2:**
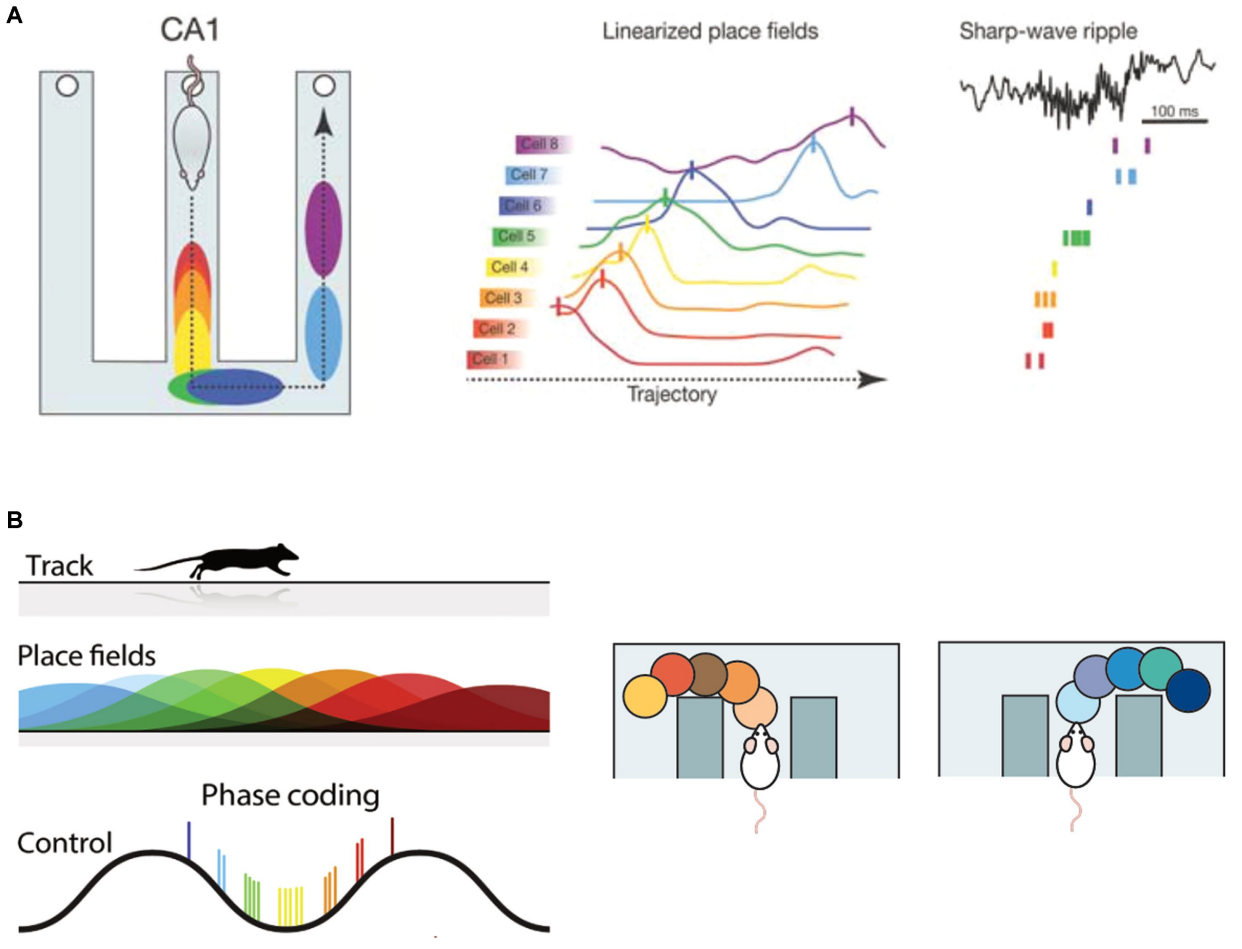
Overview of spatial sequences in the hippocampus. **(A)** Hippocampal replay: (Left) Firing of hippocampal place cells as a rat runs on a maze. The cells are successively activated as the animal traverses through their place fields (color-coded on the maze), forming a sequence. (Center) Place cells indicate spatial location by firing maximally at their preferred location (known as a place field). (Right) Sharp-wave ripples (SWRs) occur during NREM sleep or quiet wakefulness and is associated with increased hippocampal population activity. During a SWR, place cell trajectories that were experienced during wakefulness are “replayed.” Adapted with permission from Zielinski et al. (2017). **(B)** Encoding of spatial location within theta sequences. (Left) The location of the animal is encoded via a phase code of the theta oscillation, with past locations being represented on the negative phase and future locations on the positive phase of the theta oscillation. The current location is represented at the trough of the oscillation. Reprinted from Petersen and Buzsáki (2020), with permission from Elsevier. (Right) During a deliberative decision task, hippocampal population activity nested within theta sequences sweeps forward in time, representing future spatial options. Reproduced from Redish (2016) with permission from SNCSC.

Reactivation events that have a temporal sequence (for example, a sequence that corresponds to a trajectory of place fields) are said to be *replayed* (Figure 2A). Hippocampal replay and offline replay as implemented in SR-Dyna have direct parallels, since both involve the recapitulation of previously experienced events and states, respectively. Importantly, hippocampal replay is thought to serve as a substrate for memory consolidation. Impairing SWRs or prolonging them can worsen or improve task performance, respectively (Girardeau et al., 2009; Fernández-Ruiz et al., 2019). Improved performance is thought to occur by enabling the animal to visualize paths never visited before (Ólafsdóttir et al., 2015; Igata et al., 2021) and using an internal model of the environment to plan shortcuts (Gupta et al., 2010), or to recapitulate salient place field trajectories, such as the path towards a goal (Pfeiffer and Foster, 2013), which is very reminiscent of MB and SR agents. Therefore, hippocampal replay-like sequences can improve the performance of RL agents, which can in turn provide testable predictions for systems neuroscience research to better understand the neural correlates of such sequences and how they aid decision-making.

## 7. Theta sequences and prospective coding in the hippocampus

In addition to SWRs, which are a form of offline consolidation, the hippocampus also exhibits sequences during online planning. These sequences are known as theta sequences, named after the theta oscillation, a characteristic oscillation of population activity between 8–12 Hz which is observed during locomotion or during Rapid Eye Movement (REM) sleep. Theta sequences can provide important insights into understanding the *here-and-now* type of deliberative decision-making in real-world scenarios.

O’Keefe and Recce (1993) discovered that as animals traversed across a linear track, the spiking activity of place cells shifted to earlier phases of the ongoing theta oscillation. This phenomenon is known as theta phase precession and is a crucial component for the encoding of place cell sequences (Skaggs et al., 1996; O’Keefe and Burgess, 2005) (Figure 2B, left). More recently, theta sequences were shown to be directly implicated in prospective coding. Johnson and Redish (2007) recorded hippocampal CA3 activity as the animal performed a decision task on a maze and found that the decoded population activity sequentially “looked ahead” in time to the left arm of the maze, followed by the right arm (Figure 2B, right). In another study, Wikenheiser and Redish (2015) showed that theta sequences avoid paths that the animal does not take in future, and only projects forward in time to current goals. These studies laid the foundation for a role of the hippocampus in planning and decision-making.

Additional evidence of the involvement of the hippocampus in prospective coding comes from a study done by Ito et al. (2015). They examined the activity of hippocampal neurons that fire differently based on the animal’s past or future behavior, known as *splitter cells* (Frank et al., 2000; Wood et al., 2000). These splitter cells lost their selectivity upon inhibition of the thalamic nucleus reuniens, suggesting an upstream source for this type of neural representation. The nucleus reuniens is heavily innervated by the medial prefrontal cortex (mPFC) (Vertes et al., 2007), a structure known to be involved in decision-making and which shows strong coupling to hippocampal theta oscillations during decision-making tasks (Benchenane et al., 2010; Backus et al., 2016; Tamura et al., 2017; Stout et al., 2022). Yet, so far, no functional role for the indirect projection of the mPFC to the hippocampus was shown. This study demonstrated that the feedback from mPFC is crucial for the neural representations of prospective coding in the hippocampus.

In line with the role of theta sequences in prospective coding, Kay et al. (2020) found that cells in hippocampus encode future spatial trajectories on a theta cycle-by-cycle basis, suggesting important implications of theta-cycle skipping cells that are found in the nucleus reuniens (Jankowski et al., 2014), and generally towards the role of hippocampus in planning. However, the relationship between SWRs and theta sequences, and their specific roles for planning is less understood.

## 8. A broader view of hippocampal sequences

Hippocampal sequences are thought to be essential features for encoding episodic memory. This is because episodic memory is also sequential in nature. However, along with spatial details, these episodes also typically involve temporal details. In particular, understanding the neural basis of timing is important to understand memory-guided decision-making, because when we make decisions, we typically recall events that may go back to several years ago. Below, we will touch upon temporal sequences in the hippocampus and how an understanding of these sequences can inform decision-making research.

Studies by Dragoi and Tonegawa (2011, 2013), Farooq and Dragoi (2019), and Farooq et al. (2019) demonstrated that hippocampal population activity decoded sequences corresponding to locations in the environment that the animal had not visited yet, known as *preplay*, and went on to further characterize this phenomenon. The authors interpreted preplay as an organization of hippocampal cell assemblies into temporal sequences that were attributed with the content of a future novel experience, thereby laying the foundation for the sequence generation function of the hippocampus.

The discovery of time cells in the hippocampus (Pastalkova et al., 2008) showed that the hippocampus can use sequences to encode time intervals leading up to the end of a delay period. Along with this, other findings showing the evolution of hippocampal activity over hour-long intervals (Manns et al., 2007) suggest that the hippocampus utilizes sequences to represent different time scales as well.

One prevailing theory for the representation of time is known as the temporal context model (TCM) (Howard and Kahana, 2002). At the core of TCM is the idea that experience consists of current sensory input as well as recent past sensory experience weighted with exponential decay. TCM has been thought to be a candidate theory explaining the hippocampal splitter cell phenomenon (see Duvelle et al., 2023 for review). Interestingly, the mathematical framework of SR is equivalent to a generalized form of TCM for human free recall experiments (Gershman et al., 2012).

Howard et al. (2014) used a parsimonious mathematical model to implement TCM. They encoded events in time via a set of leaky integrators. Specifically, these model neurons encoded the Laplace transform of the input, which is equivalent to decomposing a signal using exponential decay functions of different rates. With this setup, they were able to show that an approximation of the inverse Laplace transform can recover the input sequence, and this property could be applied to encode different events in time or the trajectory to a given location, for example. Further analysis of time cell activity revealed a correspondence between the model neurons and experimentally observed properties of time cells, suggesting a mechanism for how the hippocampus encodes spatiotemporal information using sequences. This model was used in conjunction with the SR to implement multi-scale SR in Momennejad and Howard (2018).

In summary, spatiotemporal sequences are thought to be the neural substrates for human episodic memory that is vivid and rich with spatiotemporal and other multimodal information such as olfaction; the smell of our mother’s cooking can take us back to our childhood in a flash. The hippocampus is thus thought to bind all these features together into a coherent representation of memory. Understanding how the reward learning system utilizes the information encoded in hippocampal sequences representing such a vast diversity of information to guide decisions is an exciting direction of research for the decision-making field.

## 9. Beyond the rodent hippocampus

Extending the findings from rodent studies to humans is important to understand the mechanisms of decision-making. However, the techniques used to study the precise timing of hippocampal sequences are difficult to directly be applied to human research for various reasons. First, electrophysiological recordings are invasive, and are therefore only performed on patients who are being monitored for surgical removal of epileptic tissue. These patients often have altered brain activity and impaired decision-making, making it difficult to study what happens in a healthy individual. Having access to a good sample size of patients is an additional challenge. Second, non-invasive techniques such as fMRI are useful to study healthy individuals, but have poorer temporal resolution, making it a challenge to study hippocampal sequences such as SWRs and theta sequences which occur on the scale of milliseconds.

Despite these challenges, research in humans is catching up with the advances made in rodent spatial navigation with the demonstrations of place, grid, and time cells, using single unit recordings and fMRI (Ekstrom et al., 2003; Doeller et al., 2010; Jacobs et al., 2013; Umbach et al., 2020). In addition, hippocampal reactivation of both spatial and non-spatial representations as well as SWRs have been demonstrated in humans and are positively associated with memory performance, as known from rodent studies (Axmacher et al., 2008; Schapiro et al., 2018; Liu et al., 2019; Norman et al., 2019; Schuck and Niv, 2019; Jacobacci et al., 2020). A recent report has also shown evidence supporting hippocampal sequence generation in the human medial temporal lobe (Vaz et al., 2023).

Recent research with human subjects offers promising avenues for the role of the hippocampus in learning and decision-related activity. Using fMRI in infants, Ellis et al. (2021) showed that the hippocampus supports statistical learning from an early age. In addition, the hippocampus has been implicated in approach-avoidance decision-making (O’Neil et al., 2015; Ito and Lee, 2016) as well as in MB planning (Miller et al., 2017; Vikbladh et al., 2019), thereby demonstrating the relevance of considering hippocampal contributions to different types of decision-making, especially memory-guided decision-making, which is a rather novel area of interest (Weilbächer and Gluth, 2017; Mızrak et al., 2021).

Despite being limited by measuring vascular responses and poor spatiotemporal resolution, fMRI offers whole-brain access, which is not as easy in rodents with current techniques. This has led to deeper insights on how the hippocampus, in conjunction with the prefrontal cortex and striatum, represents abstract information during decision-making tasks, such as representations of task structure from experience in conjunction with the orbitofrontal cortex (Mızrak et al., 2021), the combination of spatial and non-spatial variables during goal-directed decision-making (Viard et al., 2011), and deliberation during value-based decision making (Bakkour et al., 2019). Ross et al. (2011) demonstrated increased functional connectivity between the hippocampus, striatum, and prefrontal regions during distinct phases of a context-dependent decision-making task, suggesting that there is extensive crosstalk between these regions, but the full spectrum of interactions and how they give rise to behavior have not been delineated yet.

Such whole-brain studies have also led to the characterization of other distinct brain network modules, such as the dorsal and ventral attention networks, the default mode network, and the visual network to name a few (Power et al., 2010).

These brain networks have confirmed that regions that were thought to work in synchrony are indeed co-modulated during tasks such as attention, memory, and decision-making. Notably, the hippocampus along with the prefrontal cortex is part of the default mode network (DMN), a network thought to be active when we are not engaged in any task but are introspecting, deliberating, or recalling past experiences (Buckner and Carroll, 2007). In addition, hippocampal SWRs are accompanied by increased cortical activation in nodes of the DMN (Kaplan et al., 2016), suggesting that SWRs and associated replay events are processes having potential brain-wide consequences. Using wide-field voltage imaging in mice, the retrosplenial cortex was shown to exhibit the highest degree and the shortest latency activation post-SWR (Abadchi et al., 2020). Linking the various nodes of the DMN with specific hypotheses about their function in decision-making would offer a wealth of knowledge about the component processes of decision-making. A better understanding of these network-level dynamics will emerge via the integration of structures hitherto ignored in the field, such as the hippocampus.

## 10. Hippocampal contributions to understanding the self and lifelong real-world decision making

The field of neuroeconomics is predominantly inundated with research in value-based decision-making, focusing on the striatum and prefrontal cortex. As the *self* embodies a person’s lifelong decision-making and experience, can a deeper and more comprehensive mapping of the interaction of hippocampus with the striatum and prefrontal regions open new horizons for scaling up the impact of neuroscience research on real-world applications for better mental health and wellbeing?

The self is at the core of our mental life, creating a continuous thread guiding decision-making over the course of a person’s lifespan and as a function of real-time and cumulative experience and context (Koban et al., 2021). From an evolutionary perspective, self-in-context representations are internal models of situations and underlying causal structures that bear on future survival and wellbeing. The self has been studied across disciplines spanning philosophy, psychology, and more recently, neuroscience (see Koban et al., 2021 for review). Autobiographical memory is at the core of the self. Other central features of the self may include feelings of agency, feelings of ownership towards the body, experiencing the self as a unit, and self-referential labeling of stimuli. A continuous sense of self can guide our real-time decision-making and behavior while providing a sense of continuous agency and identity across the lifespan.

Gallagher (2000) posited the self as an interaction between an episodic, executive minimal self and a temporally extended narrative self, which includes past memories and future intentions as well as a sense of continuity between these temporal states. This account therefore suggests that episodic memory, specifically, having a sense of time and context, is essential for the construction of the narrative self. This begs the question of linking hippocampal function to an understanding of the emergence of the self.

Koban et al. (2021) further suggest that self-in-context representations are simultaneously *generative* (i.e., allows one to simulate the consequence of potential actions), *interpretive* (i.e., enables the understanding of incoming sensory signals), *attributive* (i.e., assigns latent causes to sensory events), *instructive* (causal attributions shape what is learned from experience), *predictive* (in that they predict what one will experience in a given condition and context), and finally *motivational,* as they can mobilize cognitive, affective, and physiological systems for physical and mental well-being.

The multi-functional view of the self featured above provides important insights on how the double integration of the self and hippocampus in current neuroeconomics approaches to value-based choice can advance a real-world decision-making framework that is biological, culturally and psychologically plausible. As mentioned at the onset, the prevailing assumption in decision neuroscience and neuroeconomics is that the brain reward system encodes representations of the online expected value of stimuli and/or actions through the ventromedial prefrontal cortex (vmPFC), supplementary motor area, and the striatum (Balleine et al., 2007; Fellows and Farah, 2007; Kennerley and Walton, 2011; Lee et al., 2021; Aquino et al., 2023). The brain then is thought to make a real-time choice based on the MF and/or MB strategies to maximize future expected rewards. Inter-temporal choice is often investigated using delay-discounting functions which account for the reduction in the net present value of future outcomes.

The field of neuroscience has started to explore the neural mechanisms underlying our sense of self (see Herbert et al., 2016; Schaefer and Northoff, 2017 for review), with the self being seen as a multimodal and multiscale neural-psycho-social structure (Kotchoubey et al., 2016) only partly overlapping with brain reward systems (Northoff and Hayes, 2011; Lipsman et al., 2014), tied to contextual dynamics of temporal and spatial organization of spontaneous brain activity (Zhang et al., 2018), providing life course psychological continuity to an individual, and guiding immediate as well as long term real-world decisions (Herbert et al., 2016). Finally, it can also be linked to the environment (or social spheres) through the properties of embodiment and embeddedness (Schaefer and Northoff, 2017).

We now know that accounts of value-based decision-making, (and potentially also of the self), are incomplete without the incorporation of the hippocampus and would therefore like to usher a change in the framework of current decision-making research by advocating that the hippocampus is a crucially essential component underlying decision-making and the self.

As discussed in this review, the hippocampus can potentially implement a predictive framework of the environment, exemplified by the neural correlates of SR models. As shown by multi-scale SR models, this predictive representation can span multiple timescales. Understanding how memory and time are integrated in the brain offers an attractive avenue for understanding the relationship between the episodic memory system, the self, and decision-making, mediated by the hippocampus and the DMN, in conjunction with the striatal reward learning system. In addition to the hippocampus, interval timing is also represented in the prefrontal and parietal cortical areas and is thought to be integrated via the striatum (Leon and Shadlen, 2003; Lustig et al., 2005; Kim et al., 2013; Howard et al., 2015).

Finally, recent neuroimaging evidence sheds light on non-value signals from the hippocampus and the rest of the DMN (Bakkour et al., 2019). Together, these signals contribute to decision-making through the integration of different timescales into a person’s real-time experience and choice, which, over time, accumulates in a temporally extended representation of the self (Biderman et al., 2020). Thus, the self becomes a key thread that connects episodic decision-making with longer-term, non-value factors that are left out of classical decision models.

## 11. Discussion

Due to the multi-disciplinary nature of the decision-making field, several attractive directions of research present themselves in the concluding section of this article, having far-reaching implications not only in the fields of computational modeling, but also spatial navigation, neuroeconomics, marketing, and health. Advances in these disciplines will, in turn, provide an integrative account of the mechanisms underlying real-world decision-making along a person’s life course.

We now have powerful computational tools capable of solving a variety of “here-and-now” decision-making tasks and are beginning to understand the mechanisms behind credit assignment of future states, which evidence is important during credit assignment how belief updating is integrated into the memory system, and how this leads to changes in policy. The hippocampus is poised to serve crucial roles in these processes, making it relevant to decision-making researchers.

One exciting avenue in accounting for the role of hippocampal replay in decision-making over time lies in the domain of continual learning (CL). In CL, a model must continually learn new tasks, while maintaining its performance on previously learnt tasks. A major challenge in CL is *catastrophic forgetting*, in which performance on previously acquired tasks drops due to interference with learning of a new task. However, we know that animals can learn many different concepts throughout their lifetime without forgetting previously learnt ideas. Interestingly, using replay to counteract catastrophic forgetting is emerging as a popular approach in the field (van de Ven et al., 2020; Kowadlo et al., 2022; Stoianov et al., 2022). Specifically, models have begun to utilize *generative replay* (van de Ven et al., 2020; Stoianov et al., 2022), in which fictive experiences sampled from a generative model are replayed. This approach is computationally advantageous since it does not require extensive storage capacity to keep a record of all previous experience but is also biologically plausible since not all replay events are an exact recapitulation of past events. Stoianov et al. (2022) went one step further to show that an agent using generative replay that prioritizes “surprising” experiences can outperform CL agents using exact replay.

However, the role of theta sequences in a CL agent is less clear. SWRs and theta sequences co-exist in the hippocampus with an inverse relationship; ripples occur during so-called offline states, while theta sequences occur during real-time decision-making. Novel computational approaches can provide answers to which process is important when and for what kind of tasks. Specifically, disambiguating the exact roles that these oscillations play in the context of learning, consolidation and planning will directly be of benefit for biologically plausible modeling of neural representations and behavior. In addition, understanding the content and valence associated with hippocampal sequences and how these factors are integrated with the striatal and prefrontal systems will bring more clarity in our understanding of the meaning behind the activation of brain networks such as the DMN post-ripple.

CL models using generative replay approaches may also contribute to an understanding of autobiographical memories. By continuously encoding and replaying episodes, such agents may provide an account of events that persists through time. By combining the past with the present and prospecting about the future, this can advance the understanding of the neural correlates of the sense of self and its roles in episode-specific and lifelong learning and reward (Addis et al., 2011; Stoianov et al., 2022). In fact, a recent animal-model based study (Miller et al., 2022) provides strong support in favor of adopting a holistic approach towards decision-making research that incorporates learning, multi-scale approaches and continuity, while moving away from simplistic value-based decision-making. This will lead to better mechanistic insights into how the human self utilizes reward learning in real-world choice behaviors, which is a very promising research direction.

## 12. Conclusion

The overarching message of this article is to portray the hippocampus as a key but understudied aspect of human decision-making. By using past information in the form of memory to guide our future actions, the hippocampus could well be a core neural substrate of decision-making *per se*, and its interaction with the striatum and prefrontal regions – continuously updating and modulating the MF-MB balance, thereby impacting real-world decision-making in the *“here and now”* as well as on the long term, with the accumulation of experiences and contexts as the self, unfolding over time and across scales and dimensions (Northoff and Hayes, 2011; Koban et al., 2021; Dubé et al., 2022).

Our species has reached its current state through the evolution of a highly sophisticated brain engaging in decision-making that ranges from canonical MF and MB to something in between, with the self being one of the most distinguishing facets of human evolution. This enables real-world behavior to be adaptive to an ever more complex and dynamic immediate environment as well as to social institutions and globe-spanning digital communities. Creating a world that supports multiscale computational efficiency and resilience in human and machine is a pressing necessity (Dubé et al., 2022). While conceptual, methodological and computational challenges in integrating space, time, and memory are abundant for humans (Gershman et al., 2015) and machines (Rahwan et al., 2019), recent developments in animal and human brain research (Howard et al., 2014; Eichenbaum, 2017) are opening pathways to next-generation precision convergence science (Dubé et al., 2022) that not only builds upon but goes beyond the convergence of -omics, engineering and clinical sciences that have been found life-saving, for instance in the context of cancer and the COVID-19 pandemic (Sharp and Langer, 2011). The time is ripe for a world-saving convergence between neuroscience, neuroeconomics, management, and related disciplinary research that examines multiscale mechanisms in and between humans, machines, and human-made systems to converge in novel ways to accelerate real world solutions at scale.

## Author contributions

DM performed the literature review and wrote the first draft of the manuscript. DM and LD wrote sections of the manuscript. All authors contributed to the article and approved the submitted version.

## Funding

DM is funded by a doctoral training award by the Fonds de Recherche du Québec, Santé. LD is funded by the Driving adaptive versus materialistic consumption to benefit consumers and marketers. Grant # SSHRC 435-2020-1136. Implementing Smart Cities Interventions to Build Healthy Cities. Healthy Cities Training. Grant # CIHR-NSERC-SSHRC/Guelph 02083-000.

## Conflict of interest

The authors declare that the research was conducted in the absence of any commercial or financial relationships that could be construed as a potential conflict of interest.

## Publisher’s note

All claims expressed in this article are solely those of the authors and do not necessarily represent those of their affiliated organizations, or those of the publisher, the editors and the reviewers. Any product that may be evaluated in this article, or claim that may be made by its manufacturer, is not guaranteed or endorsed by the publisher.
